# GSK3ß inhibitor CHIR 99021 modulates cerebral organoid development through dose-dependent regulation of apoptosis, proliferation, differentiation and migration

**DOI:** 10.1371/journal.pone.0251173

**Published:** 2021-05-05

**Authors:** Chloe Delepine, Vincent A. Pham, Hayley W. S. Tsang, Mriganka Sur

**Affiliations:** 1 Picower Institute for Learning and Memory, Massachusetts Institute of Technology, Cambridge, Massachusetts, United States of America; 2 Department of Brain and Cognitive Sciences, Massachusetts Institute of Technology, Cambridge, Massachusetts, United States of America; 3 Simons Center for the Social Brain, Massachusetts Institute of Technology, Cambridge, Massachusetts, United States of America; Lewis Katz School of Medicine at Temple University, UNITED STATES

## Abstract

Cerebral organoids generated from human pluripotent stem cells (hiPSCs) are unique in their ability to recapitulate human-specific neurodevelopmental events. They are capable of modeling the human brain and its cell composition, including human-specific progenitor cell types; ordered laminar compartments; and both cell-specific transcriptional signatures and the broader telencephalic transcriptional landscape. The serine/threonine kinase, GSK3β, plays a critical role in neurodevelopment, controlling processes as varied as neurogenesis, morphological changes, polarization, and migration. In the generation of cerebral organoids, inhibition of GSK3β at low doses has been used to increase organoid size and decrease necrotic core. However, little is known of the effects of GSK3β inhibition on organoid development. Here, we demonstrate that while low dose of GSK3β inhibitor CHIR 99021 increases organoid size, higher dose actually reduces organoid size; with the highest dose arresting organoid growth. To examine the mechanisms that may contribute to the phenotypic size differences observed in these treatment groups, we show that low dose of CHIR 99021 increases cell survival, neural progenitor cell proliferation and neuronal migration. A higher dose, however, decreases not only apoptosis but also proliferation, and arrests neural differentiation, enriching the pool of neuroepithelial cells, and decreasing the pools of early neuronal progenitors and neurons. These results reveal new mechanisms of the pleiotropic effects of GSK3β during organoid development, providing essential information for the improvement of organoid production and ultimately shedding light on the mechanisms of embryonic brain development.

## Introduction

Glycogen synthase kinase 3 beta (GSK3β) is a serine/threonine kinase, which has an extended 13 amino acid loop by the catalytic domain [1]. During neurodevelopment, GSK3β controls the level, binding, and localization of transcription factors (such as CREB, Nfat, Neurogenin 2, SMAD1, and β-catenin) as well as the activity of cytoskeletal proteins involved in migration and axonal growth and guidance. Altogether, GSK3β coordinates a wide range of signaling processes guiding neurodevelopmental events that include neurogenesis; structural changes and cell advancement; polarization and orientation; and migration [2]. The canonical Wnt/β-catenin pathway is the best characterized pathway mediated by GSK3β. The absence of Wnt facilitates GSK3β-mediated phosphorylation of β-catenin, which leads to its ubiquitination and proteasome-mediated degradation [3]. In the presence of Wnt, or inhibition of GSK3β, the consequent reduction of β-catenin phosphorylation and degradation allows β-catenin to accumulate in the cytoplasm, associated with its translocation to the nucleus and the transcriptional regulation of Wnt target genes. Inhibition of GSK3β stabilizes β-catenin and in turn activates the Wnt pathway, independently of Wnt ligand-receptor interactions.

Understanding human brain development with little access to fetal tissues remains a major challenge in neuroscience. *In vitro* cultures of cerebral organoids derived from human pluripotent stem cells have therefore raised the promise of effectively studying and modeling human brain development. However, protocols for brain organoid production are limited by our partial knowledge of the mechanisms of brain development. In the past decade the field has developed both self-organizing protocols and more recent directed protocols for the production of brain organoids encompassing broad and specific regional identity, respectively; however many limitations remain, including difficulties in the maintenance of healthy cells throughout long-term cultures, and efficient and reliable determination of cell fate and identity [4]. Inhibition of GSK3β by CHIR 99021 to promote cell survival or trigger regionalization in brain organoids has been suggested in several recent studies [5–10]; however its multiple effects on organoid development are not well characterized. Here, we demonstrate that the inhibition of GSK3β using high and low dosages of CHIR 99021 during neuronal differentiation of hiPSC-derived organoids elicits differential effects on their growth and cellular phenotypes. Understanding how the GSK3β inhibition affects cerebral organoid production not only helps improve cerebral organoid protocols but also reveals human neurodevelopmental processes.

## Results

### GSK3β inhibition during neuronal differentiation affects organoid growth

**Fig 1:**
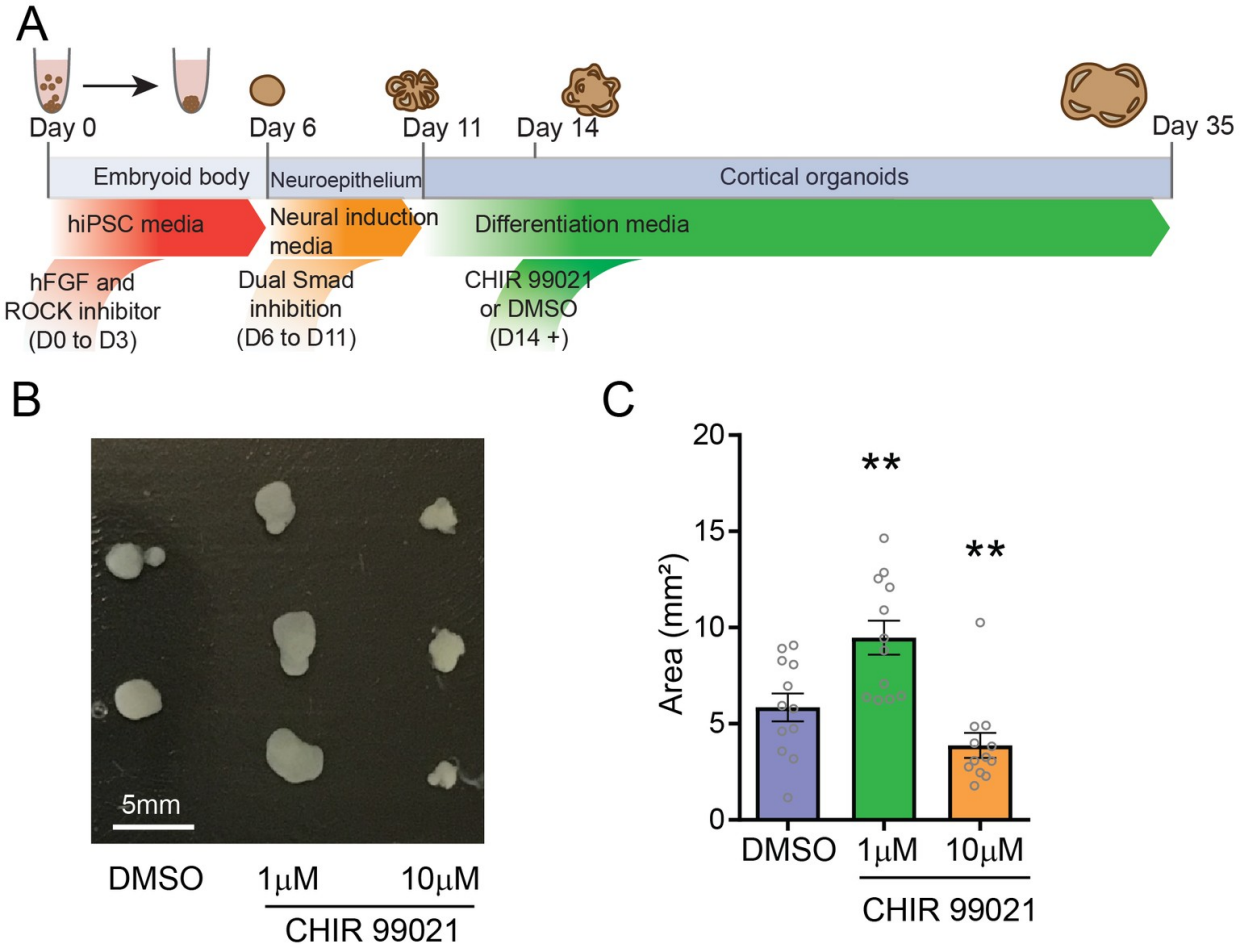
GSK3β inhibition by CHIR 99021 during neuronal differentiation affects neurodevelopmental signaling pathways and organoid growth. **A**. Schematic of organoid production protocol. Dual SMAD inhibition was performed during the neural induction step. GSK3β inhibitor CHIR 99021 (1μM, 10μM or 50μM), or vehicle DMSO, was added to the culture media throughout neuronal differentiation (day 14 and onward). **B**. Representative organoids at day 35 of organoid development; organoids treated with 50μM CHIR 99021 failed to grow. **C**. Quantification of organoid size. CHIR 99021 treatment had a dose-dependent effect on organoid size (DMSO: 5.856 ± 0.7229, n = 12; 1μM: 9.472 ± 0.8797, n = 12; 10μM: 3.294 ± 0.3062, n = 11; DMSO vs 1μM, **: p-value = 0.0044; DMSO vs 10μM, **: p-value = 0.0047; 1μM vs 10μM, p-value<0.0001; unpaired t-tests). Bar graphs represent mean ± SEM.

To examine the graded effects of GSK3β inhibition, we treated organoids with three doses of CHIR 99021—1μM, 10 μM and 50μM—and a DMSO vehicle. Organoids were chronically treated from day 15 of *in vitro* production, following the neural induction stage and concurrent with the neural differentiation stage (Fig 1A). Size was measured at day 35 for all treatment doses, as our previous work indicated that 5 weeks of organoid differentiation is the ideal time point to capture the laminar composition reflective of early cortical development, where the subventricular zone is overrepresented relative to the nascent cortical plate [11]. We found that while organoid size increased with 1μM treatment (1.6-fold increase), higher concentrations led to a visible reduction in size (10μM: 1.8-fold decrease) or even growth arrest (Fig 1B and 1C). Organoids treated with the highest dose (50μM) failed to grow soon after treatment began (Fig 1B and 1C).

### GSK3β inhibition during organoid neuronal differentiation decreases apoptosis, and at high dose but not low dose decreases proliferation

**Fig 2:**
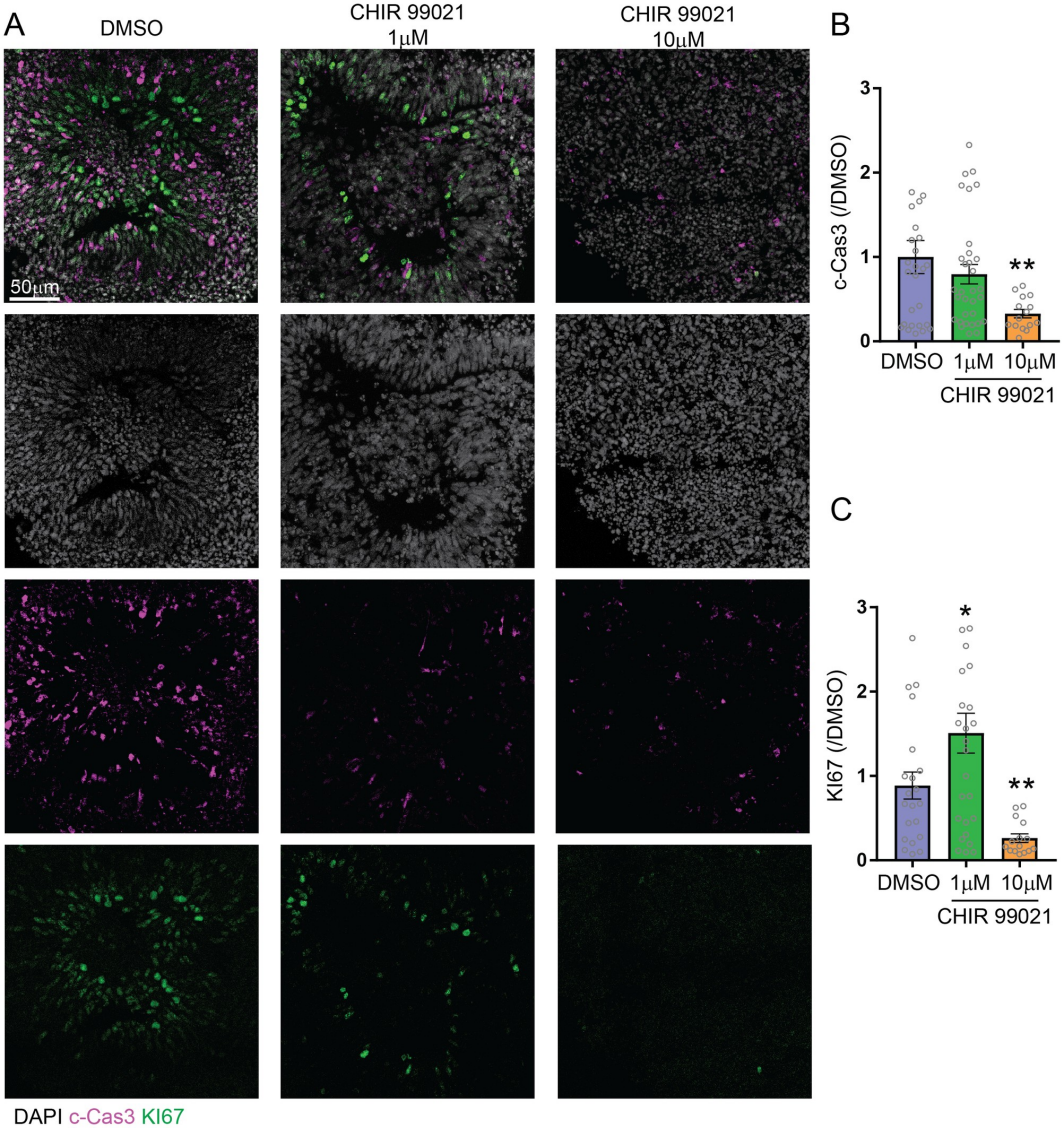
GSK3β inhibition by CHIR 99021 decreases apoptosis, and at high dose, but not low dose, decreases proliferation. **A**. Representative images of immunostained organoids. The apoptosis marker cleaved caspase 3 (c-Cas3) and proliferation marker KI67 were immunolabeled. **B**. Quantification of the c-Cas3 or KI67 positive areas normalized by the DAPI positive area for each organoid (c-Cas3: DMSO: 1 ± 0.1972, n = 27; 1μM: 0.7968 ± 0.1147, n = 32; 10μM: 0.3282 ± 0.04979, n = 15; DMSO vs 1μM, p-value = 0.3592, DMSO vs 10μM, *: p-value = 0.0185. KI67: DMSO: 0.8857 ± 0.1606, n = 21; 1μM: 1.508 ± 0.2367, n = 27; 10μM: 0.2625 ± 0.05119, n = 15; DMSO vs 1μM, *: p-value = 0.0463, t-test; DMSO vs μM, **: p-value = 0.0031; unpaired t-test). Bar graphs represent mean ± SEM.

To explore the possibility that the increase in organoid size associated with low dose treatment (1μM) and that the decrease in size associated with higher dose (10μM) may be the result of increased apoptosis, and/or decreased proliferation, we stained organoid slices with the apoptotic marker cleaved-caspase3 (c-Cas3), and the proliferation marker KI67. We found that at low dose of CHIR 99021 (1μM), the expression of c-Cas3 was reduced (Fig 2A and 2B; 1.3-fold reduction) and the expression of KI67 was increased (Fig 2A and 2C; 1.5-fold increase) compared to control, suggesting that decreased apoptosis and increased proliferation both participated in the increase in organoid growth at low dosage. At a higher dose of CHIR 99021 treatment (10μM), expression of c-Cas3 was further reduced (Fig 2A and 2B; 3-fold reduction) but, in contrast to the lower dose, expression of KI67 was also significantly reduced (Fig 2A and 2C; 1.6-fold reduction). This result suggests that both proliferation and apoptosis were reduced at the higher dose of CHIR 99021 (10μM) exposure.

### GSK3β inhibition by CHIR 99021 affects neuronal differentiation of neuroepithelium in a dose dependent manner

**Fig 3:**
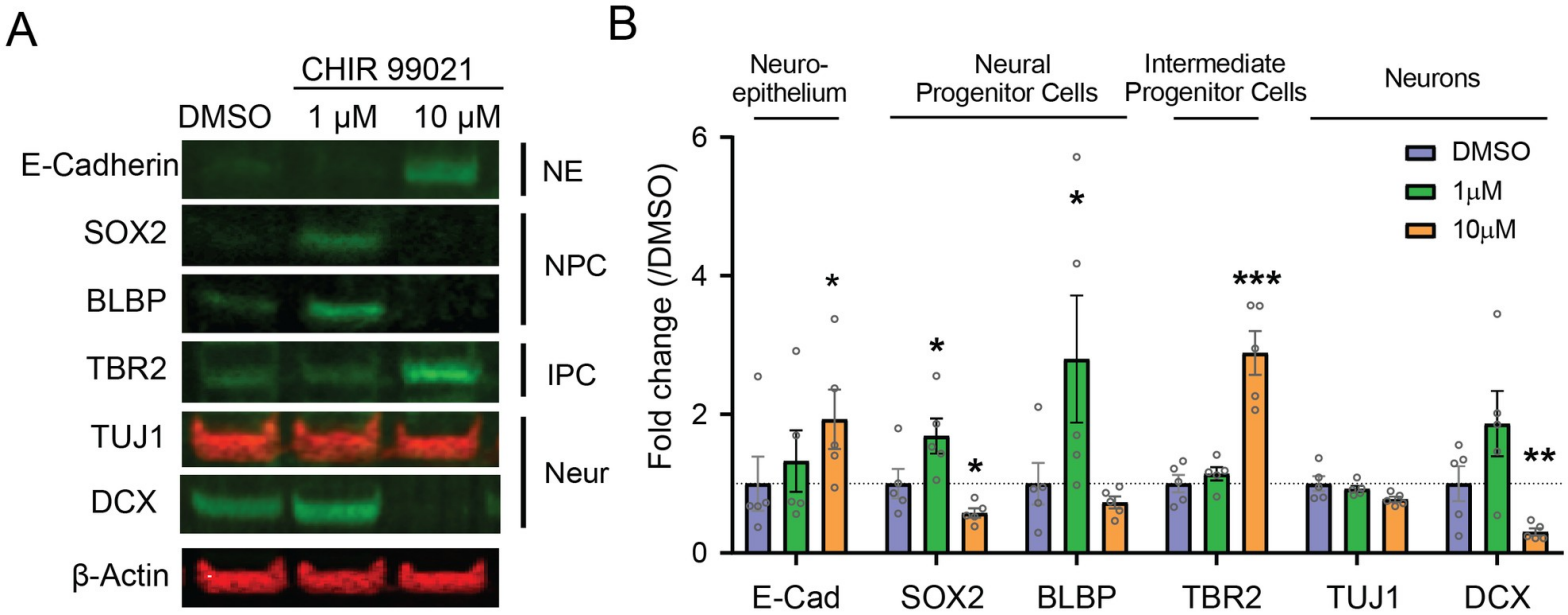
GSK3β inhibition by CHIR 99021 affects neuronal differentiation of neuroepithelium in a dose dependent manner-Western Blots. **A**. Western Blot bands and **B**. quantification of neuroepithelium marker E-cadherin (E-Cad), neural progenitor cells (NPCs) marker SOX2, radial glia marker BLBP, intermediate progenitor marker TBR2, and neuronal markers TUJ1 and DCX, relative to DMSO control (n = 5 batches of organoid production for the three groups. E-cadherin: DMSO: 1 ± 0.0891; 1μM: 0.9285 ± 0.2585, p-value = 0.2881; 10μM: 1.819 ± 0.5351, *: p-value = 0.0286; SOX2: DMSO: 1 ± 0.09242; 1μM: 1.472 ± 0.1668, *:p-value = 0.0125; 10μM: 0.5228 ± 0.05321, *:p-value = 0.0405; BLBP: DMSO: 1 ± 0.01525; 1μM: 2.069 ± 0.7181, *:p-value = 0.0286; 10μM: 0.6944 ± 0.1048, p-value = 0.8825; TBR2: DMSO: 1 ± 0.1263; 1μM: 1.141 ± 0.09633, p-value = 0.4000; 10μM: 2.884 ± 0.3168, ***: p-value = 0.0006; TUJ1: DMSO: 1 ± 0.1075; 1μM: 0.9262 ± 0.04413, p-value = 0.5429; 10μM: 0.7731 ± 0.0886, p-value = 0.0823; DCX: DMSO: 1 ± 0.2530; 1μM: 1.864 ± 0.4717, p-value = 0.1449; 10μM: -1.516 ± 0.0886, p-value = 0.0044, unpaired t-tests).

**Fig 4:**
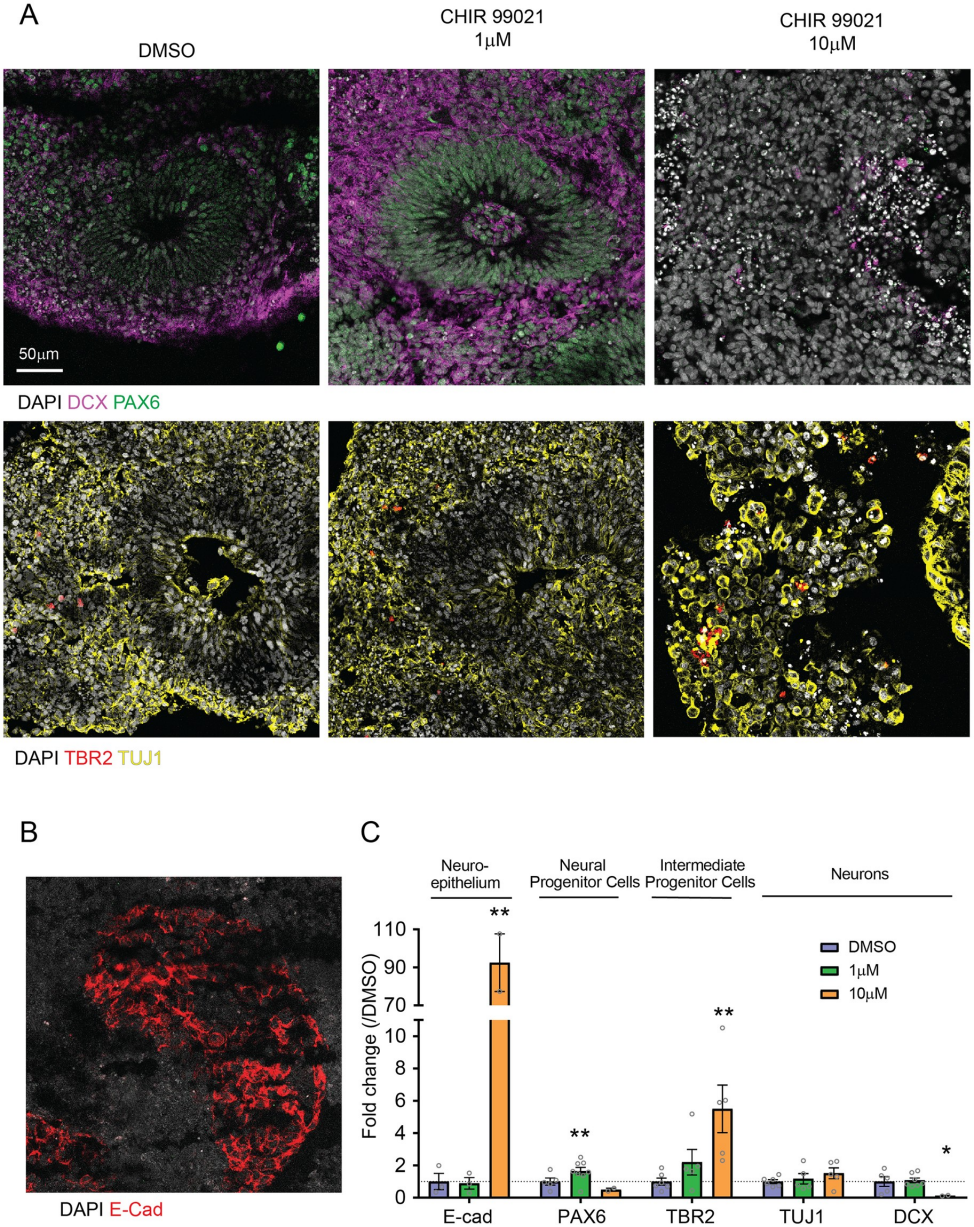
GSK3β inhibition by CHIR 99021 affects neuronal differentiation of neuroepithelium in a dose dependent manner-IHC. **A**. Representative images of immune-stained organoids for detection of NPC marker PAX6, neuronal markers DCX and TUJ1, intermediate progenitor marker TBR2, and **B**. neuroepithelial marker E-cadherin. **C**. Quantification of the cell type marker positive areas normalized by the DAPI positive area for each organoid, and relative to DMSO organoids (E-Cad: DMSO: 1 ± 0.5046, n = 3; 1μM: 0.8878 ± 0.3554, n = 3; 10μM: 92.47± 11.34, n = 2; DMSO vs 1μM, **: p-value = 0.8646; DMSO vs 10μM, **: p-value = 0.0040. PAX6: DMSO: 1 ± 0.2189, n = 5; 1μm: 1.834 ± 0.1585, n = 7; 10μM: 0.5 ± 0.09146, n = 2; DMSO vs 1μM, **: p-value = 0.0099; DMSO vs 10μM, p-value = 0.2339. TBR2: DMSO: 1 ± 0.2185, n = 6; 1μM: 2.2 ± 0.7904, n = 5; 10μM: 5.5 ± 1.471, n = 5; DMSO vs 1μM, p-value = 0.1456; DMSO vs 10μM, **: p-value = 0.0087; DCX: DMSO: 1 ± 0.3027, n = 5; 1μM: 1.078 ± 0.1477, n = 7; 10μM: 0.1283± 0.2005, n = 2; DMSO vs 1μM, p-value = 0.8046; DMSO vs 10μM, *: p-value = 0.0449; TUJ1: DMSO: 1 ± 0.1175, n = 6; 1μM: 1.17 ± 0.3217, n = 5; 10μM: 1.512 ± 0.3407, n = 5; DMSO vs 1μM, p-value = 0.6026; DMSO vs 10μM, p-value = 0.1583; unpaired t-test.). Bar graphs represent mean ± SEM.

To better understand the effect of CHIR 99021 treatment on organoid differentiation, we then probed for the expression of cell-type markers in organoids treated with CHIR 99021, using Western Blot (WB) (Fig 3), and immunohistochemistry (IHC) (Fig 4). Organoids treated with low dose of CHIR 99021 (1μM) showed increased levels of neural progenitor cell (NPC) markers SOX2 (WB, 1.5-fold increase) and PAX6 (IHC, 1.8-fold increase), and of radial glia marker BLBP (WB, 2.1-fold increase). No change was observed for levels of neuroepithelium marker E-cadherin and neuronal markers TUJ1 and DCX. In contrast, organoids treated with 10μM CHIR 99021 showed increased levels of E-cadherin (WB and IHC, 1.8-fold and 92-fold increase) and of intermediate progenitor marker TBR2 (2.9-fold increase), decreased levels of NPC markers SOX2 (WB, 1.9-fold decrease) and PAX6 (IHC, 2-fold decrease), and decreased levels of neuronal marker DCX (IHC, 7.8-fold decrease). This suggests that GSK3β signaling modulates neuroepithelium differentiation in a dose dependent manner.

### GSK3β inhibition by low dose CHIR 99021 increases neuronal migration

**Fig 5:**
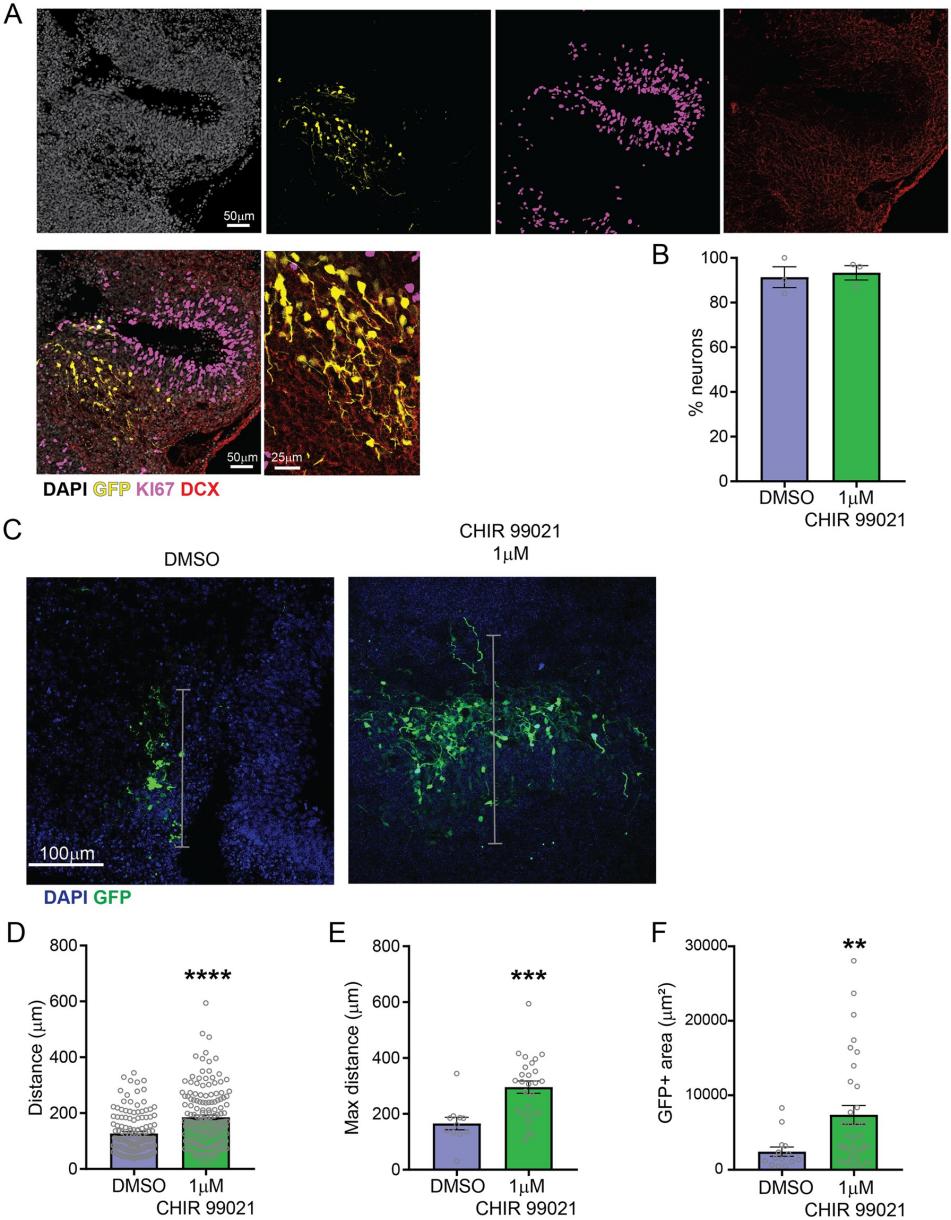
GSK3β inhibition by CHIR 99021 increased neuronal migration. **A**. Representative images of GFP expressing cells 7 days after injection and electroporation of the GFP expression plasmid, with immunostaining of proliferative cell marker KI67 and neuronal marker DCX. **B**. Quantification of the percentage of neurons (DCX+, KI67- cells) (DMSO: 91.33% ± 8.083, n = 3; 1μM: 93.33% ± 5.508, n = 3; p-value = 0.7411, unpaired t-test) **C**. GFP positive area, distance and maximum distance (in gray) from injection ventricle were measured. **D**. Quantification of cell distance from injected ventricle (DMSO: 127.2±6.189, n = 135; 1μM: 184.9±8.041, n = 157; ****: p-value<0.0001, Welch’s t-test). **E**. Quantification of maximum distance from injected ventricle (DMSO: 165.4±22.58, n = 11; 1μM: 295.5±21.81, n = 26; ***: p-value = 0.0003, Welch’s t-test). **F**. Quantification of area occupied by GFP positive cells (DMSO: 2435±616.5, n = 14; 1μM: 7379±1285, n = 33; **: p-value = 0.0011, Welch’s t-test). Bar graphs represent mean ± SEM.

During cortical development, newly born neurons migrate radially from the ventricular zone to the cortical plate. We then sought to measure the effect of a low dose treatment on neuronal migration. To obtain a sparse labeling of neurons and measure the distance they travel in a certain interval of time, we injected a GFP expression plasmid in the ventricles and electroporated the ventricular zones of organoids at day 40 of development. Seven days after the electroporation, GFP expression and distance from the injection site were analyzed. As measured by colocalization with proliferative cell marker KI67 and neuronal cell marker DCX, GFP-positive cells at that time were mostly neurons (Fig 5A and 5B; DMSO: 91.33%, CHIR 1μM: 93.33%). GFP-positive neurons (as defined by their morphology) were found at greater distance from the injection site in organoids treated with 1μM of CHIR 99021 than in controls (Fig 5C–5E; 1.8-fold increase), indicating greater migration which may in turn contribute to the increase in organoid size. Moreover, organoids treated with 1μM of CHIR 99021 showed higher proportion of cells expressing GFP than controls, as measured by the GFP positive area (Fig 5C and 5F; 3-fold increase), likely as a consequence of the increased progenitor proliferation and neurogenesis described above.

## Discussion

We found that GSK3β inhibition by CHIR 99021 during the neuronal differentiation stage affected cerebral organoid development in a dose-dependent manner. At low dose, we observed that apoptosis was reduced and that differentiation of the neuroepithelium into proliferative early neural progenitor cells was increased. Given that the expression levels of neuronal markers were similar in low dose treated organoids and controls, neurogenesis seemed relatively reduced. Therefore, at low dose, CHIR 99021 promotes cerebral organoid growth by increasing cell survival and neuronal progenitor proliferation. At higher dose, we observed that even though apoptosis was reduced, these organoids were smaller than those treated at low dose when observed at the same time point. They also presented a reduction in proliferation and differentiation of the neuroepithelium. We also noted a strong effect on the intermediate progenitor cell population. This suggests that at higher doses, CHIR 99021 prevents cerebral organoid development by triggering proliferation and differentiation arrests, despite increased cell survival.

We observed an increase in organoid size associated with low dose CHIR 99021 treatment (1μM), a decrease in size associated with higher dose (10μM) and total arrest of growth at 50μM, and therefore we explored levels of apoptosis, proliferation and differentiation in the treated organoids. We observed a reduction of apoptosis, as measured by cleaved caspase 3 levels, for both 1μM and 10μM CHIR 99021 treatment. Consistently, previous studies have demonstrated that inhibition of GSK3β with 1μM CHIR 99021 during the neural induction stage of organoid development led to an increase in organoid size [7,9] and a decrease in necrotic core, as quantified by cleaved-caspase3 positive cells [7]. This is consistent with reports that Wnt signaling blocks cell death pathways, including by upregulation of SGK1 that, in turn, negatively regulates the pro-apoptotic Forkhead box O3a (FOXO3a) transcription factor [12]. Several studies have implicated GSK3β in neural progenitor proliferation [8,13]. Deletions of the α and β isoforms of GSK3 in mouse are associated with massive hyperproliferation of neural progenitors [13]. We observe an increase in proliferation, as measured by levels of nuclear proliferation protein KI67, in organoids treated with the low dose of CHIR 99021 (1μM). Similarly, quantification of cell type markers indicated an increase in both neural progenitor cell and radial glia markers compared to controls.

In contrast, we observed a decrease in proliferative cells for the higher dose (10μM) of CHIR 99021 treatment. At this higher dose treatment, we observed an increased expression of neuroepithelium marker E-cadherin, and a decreased expression of neuronal marker DCX. Consistent with these findings, a short pulse (3 days) of a 3μM CHIR 99021 treatment at the beginning of the neural differentiation stage of organoid development was shown to increase lateral expansion of the neuroepithelium, increase forebrain specification, and delay neuronal maturation [5]. Intriguingly, at the higher dose (10μM) of CHIR 99021 treatment we observed a decrease of early progenitor markers (SOX2, PAX6) contrasted by a strong increase in intermediate progenitor marker (TBR2). This is consistent with a recent report that GSK3β inhibition by CHIR 99021 may have a differential effect on neuronal progenitor subtypes [9].

Another interesting and pleiotropic effect of CHIR 99021 on brain organoid development is its influence on dorsal forebrain identity. Chronic treatment with CHIR 99021, starting at the beginning of organoid development, was associated with decreased expression of forebrain marker FoxG1 at day 50 of organoid development [9]. Similarly, in association with BMP4, it was shown to generate 3D choroid plexus tissue (dorsomedial telencephalon) and to decreased FoxG1 expression at day 35 of organoid production [10]. In contrast, a short pulse (3 days) of a 3μM CHIR 99021 treatment starting at a later stage of organoid development (after the initial step of neural fate induction and at the beginning of the neural differentiation stage of organoid development) was shown to increase forebrain specification [5]. Similarly, in our hands, chronic CHIR 99021 treatment of organoids starting after the initial step of neural fate induction was associated with increased dorsal forebrain identity, as we observed an increase of dorsal forebrain markers PAX6 (at low dose of CHIR 99021) and TBR2 (at higher dose of CHIR 99021). This suggest that GSK3β inhibition by CHIR 99021 during the early neural induction step of brain organoid culture inhibits dorsal forebrain identity, while during the later neuronal differentiation step, it promotes dorsal forebrain identity.

Finally, we demonstrated that GSK3β inhibition increased neuronal migration distance in 1μM CHIR 99021 treated organoids. The Wnt/β-catenin pathway has been involved in neuronal migration and cortical layers specification [6]. Moreover, the migration signal Reelin triggers the inhibition of GSK3β in neuronal growth cones, in turn facilitating migration through microtubule stabilization [14]. Interestingly, forebrain organoids treated with CHIR 99021 for 20 days at a later stage of organoid development were shown to display altered lamination with abnormal co-expression of upper- and deep-layer markers [6] suggesting that GSK3β regulates neuronal migration at different stages of development.

Our study reveals key insights into the complex effects of GSK3β inhibition by CHIR 99021 during the development of brain organoids. Despite its limitations, including the use of a single hiPSC line, this work provides important knowledge for the optimization of brain organoid production protocols and, in augmenting current protocols, would expand their use to fill in gaps in human embryonic development studies using human fetal tissues. Pharmacological inhibition of GSK3β in organoid models of neurodevelopmental disorders has been shown to rescue cellular disease associated phenotypes [15,16], highlighting the importance of studying the complex roles of GSK3β to understand and treat disorders of brain development.

## Materials and methods

### Maintenance of hiPSCs

Human induced pluripotent stem cells (hiPSCs) derived from an apparently healthy control individual (Coriell Institute biobank GM23279) were used in this study. The iPSC line was primarily and routinely quality-controlled by karyotyping (G-band), mycoplasma testing and pluripotency testing (initially by teratoma test then routinely by immunostaining for pluripotency markers). Cell culture quality check and authentication were performed every ten iPSC passages. Cells were cultured in iPSC medium, consisting of DMEM:F12 (ThermoFisher) supplemented with 20% KnockOut Serum Replacement (ThermoFisher), 1X penicillin/streptomycin (ThermoFisher), 1X GlutaMAX (ThermoFisher), 1X 2-mercaptoenthanol (BioRad), and 10 ng/ml FGF-2 (Peprotech). Culture medium was changed daily. hiPSCs were passaged every week onto 6-well plates, coated with 0.1% gelatin (EMD Millipore) and pre-seeded with irradiated CF1 mouse embryonic fibroblasts (MEF, MTI-GlobalStem) at a density of 200,000 cells/well. hiPSC colonies were lifted from culture plate by treatment of 2.5 mg/ml collagenase, type IV (ThermoFisher) for 1 hr and were further dissociated into smaller colonies by manual trituration before being seeded onto feeder layer of MEF.

### Organoid culture

Human iPSC derived organoids were generated as described previously (Feldman et al. 2016). Briefly, iPSCs were detached from irradiated MEFs and plated at 9x10^4^ cells per well of an ultralow attachment 96-well plate (Corning) in iPSC medium, as described above, supplemented with FGF-2 (4ng/mL) and ROCK inhibitor (50μM; Y-27632, Tocris) (Day 0). Embryoid bodies (EBs) were subsequently transferred (day 6) to an ultra-low attachment 24-well plate (Corning) and cultured in neural induction medium, consisting of 1% N2 supplement (Invitrogen), 1% GlutaMAX (Invitrogen), 1% non-essential amino acids (NEAA, Invitrogen), 5μg/mL heparin (Sigma), and DMEM/F12 (Invitrogen), supplemented with 10μM SB431542 (Tocris Bioscience) and 1μM dorsomorphin (Stemgent). Dual SMAD-inhibition through dorsomorphin and SB431542 supplementation was performed to reduce heterogeneity and to induce dorsal patterning in the EBs during neural induction (day 7 to day 11). EBs were embedded in Matrigel (Corning) droplets on day 11 and transferred to neural differentiation medium, consisting of equal parts DMEM/F12 and Neurobasal (Invitrogen), 0.5% N2 supplement, 1% GlutaMAX, 0.5% NEAA, 100μM 2-mercaptoethanol, insulin, and 1% Pen/Strep (Invitrogen) supplemented with 1% B27 without vitamin A (Gibco, Life Technologies). On day 15, embedded EBs were transferred to a shaker and grown in neural differentiation media supplemented with B27 with vitamin A (Gibco, Life Technologies). For pharmacological inhibition of GSK3β, CHIR 99021 (Cellagen Technology) was diluted from 10μM stock to the appropriate concentration directly in neural differentiation media. Organoids were chronically treated thereafter to day 35.

### Cryosectioning and immunohistochemistry

Organoids were fixed on day 35 by a 30min incubation in 4% paraformaldehyde solution. Fixed organoids were incubated in 20% sucrose solution overnight at 4°C, followed by incubation in 30% sucrose for 3 hours before embedding and freezing in optimal cutting temperature (O.C.T) medium. Frozen organoid tissue was sliced into 20μm sections using a cryostat. Alternatively, for immunolabeling of E-cadherin, organoids embedded in O.C.T. were flash frozen at day 35, cryosectioned and fixed in 4% paraformaldehyde solution for 15min. Permeabilization/blocking was performed using 3% BSA/0.1% TX100 in TBS. Incubation of sections from cerebral organoids in primary antibodies solution was performed overnight at 4°C and in secondary antibodies solution at room temperature for 1 hour (Alexa Fluor, Molecular Probes). The following primary antibodies were used: cleaved caspase 3 (Cell Signaling, #9661, 1:200), KI67 (BD Biosciences, #550609, 1:200), E-cadherin (Cell Signaling, #3195, 1:200); DCX (Aves Labs, AB_2313540, 1:500); PAX6 (BD Biosciences, #561462, 1:200). Coverslips were affixed with ProLong Gold antifade reagent with DAPI (Life Technologies) and z-stacks were acquired using a Leica TCS SP8 confocal microscope. Analysis was performed in ImageJ. To estimate the proportion of cells expressing each marker, the positive area of detected marker was quantified and normalized by the DAPI positive area. Analysis was performed on the full organoid section; representative images show magnified cropped area for better visualization of the immunostaining. For the analysis of the migration experiments, distances were measured between the GFP labeled cell soma and the border of the nearest ventricle-like cavity (localized in the DAPI channel).

### Protein extraction and Western Blot

Six to fifteen organoids for each treatment were collected on day 35 in RIPA buffer (ThermoFisher), supplemented with an EDTA-free protease inhibitor cocktail (cOmplete, Sigma) and a phosphatase inhibitor cocktail (PhosSTOP, Roche) per respective manufacturer’s protocol. Samples were lysed through shearing force with the FastPrep-24 5G bead beating grinder and lysis system (MP Biomedicals). Overall sample protein levels were measured with the Pierce BCA Protein Assay Kit (Thermofisher) against a prediluted Bovine Serum Albumin standard (ThermoFisher) and subsequently normalized to 1μg/μL. Following normalization, 20μg of protein per sample were resolved by regular SDS–PAGE and transferred to polyvinylidene difluoride (PVDF) membranes (for compatibility with fluorescent secondary antibodies) with the iBlot 2 dry transfer system per manufacturer’s protocol (ThermoFisher). Membranes were then subjected to sequential lateral flow (SLF) of immunodetection reagents (blocking, antibody, and washing solutions) with the automated iBind Western System (ThermoFisher). The following primary antibodies were used: E-Cadherin (Cell Signaling, 1:750), TBR2/Eomes (Abcam, 1:750), TUJ1/Tubulin beta III (EMD Millipore, 1:750), SOX2 (Cell Signaling, 1:750), and BLBP (EMD Millipore, 1:500). Primary antibodies were detected with species-appropriate IRDye 680LT (1:4000) and IRDye 800CW (1:3000) secondary antibodies (LI-COR Biosciences). Blotted membranes were imaged on a LI-COR Odyssey imager. Band signals were quantitatively measured with ImageJ.

### Plasmid electroporation

Organoids were electroporated with a GFP mutant Venus construct (pCAGIG-Venus, Mellios et al. 2018) at day 40 of organoid production. Plasmids DNA solution (0.5μg/μl) was mixed with Fast Green dye (0.1%) and injected into the organoids using a pulled-glass capillary microelectrode. Successful injection was confirmed by the visualization of Fast Green dye inside the injected organoid. Immediately after DNA injection, four 50-ms electrical pulses (40V) were applied at 1s intervals using a 5mm electrode and an electroporator (EM830, BTX). The organoids were returned to the incubator after electroporation. After 7 days, the electroporated organoids were fixed, cryosectioned and imaged as described above.

### Statistical methods

Statistical analysis was performed using GraphPad Prism 7.05. All statistical tests were two-tailed unpaired t-test. Welch’s correction was performed when variances were significantly different (p <0.05) as specify in the figure legend. Mean, standard error of the mean (SEM), number of replicates, exact p-value and statistical test are reported in the figure legends. Graphs represent all single data points as well as summary statistics (mean and SEM).

## Supporting information

S1 File Raw original images, Western Blot. (PDF)
