# Supplementary figures and images for: GSK3ß inhibitor CHIR 99021 modulates cerebral organoid development through dose-dependent regulation of apoptosis, proliferation, differentiation and migration
Source: PLoS One. 2021 May 5;16(5):e0251173. doi: 10.1371/journal.pone.0251173 (PMC8099055; doi:10.1371/journal.pone.0251173)

Representative blot Fig3

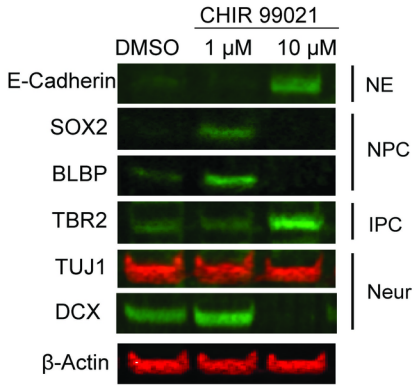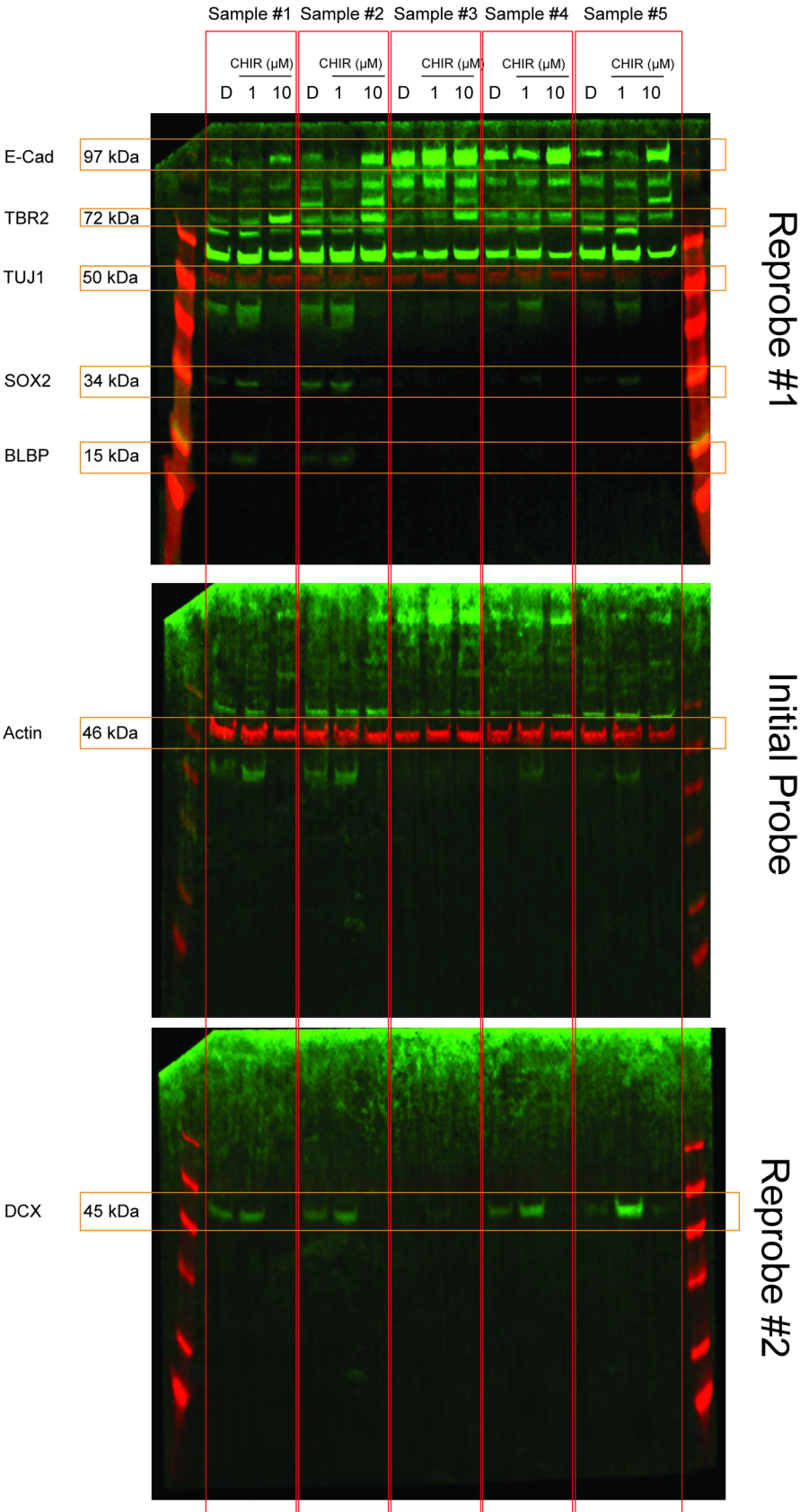

Supplement: S1 File — (PDF) [file pone.0251173.s001.pdf]
